# A Brief Review on Cerium Oxide (CeO_2_NPs)-Based Scaffolds: Recent Advances in Wound Healing Applications

**DOI:** 10.3390/mi14040865

**Published:** 2023-04-17

**Authors:** Ishita Allu, Ajay Kumar Sahi, Pooja Kumari, Karunya Sakhile, Alina Sionkowska, Shravanya Gundu

**Affiliations:** 1 Department of Biomedical Engineering, University College of Engineering (UCE), Osmania University, Hyderabad 500007, Telangana, India; 2 Faculty of Chemistry, Nicolaus Copernicus University in Torun, Jurija Gagarina 11, 87-100 Toruń, Poland; 3 Tissue Engineering and Biomicrofluidics Laboratory, School of Biomedical Engineering, Indian Institute of Technology (Banaras Hindu University), Varanasi 221005, Uttar Pradesh, India; 4 Department of Mechanical & Industrial Engineering, National University of Science and Technology, Muscat 2322, Oman; 5 Faculty of Health Sciences, Calisia University, Nowy Świat 4, 62-800 Kalisz, Poland

**Keywords:** wound healing, cerium oxide, skin, scaffolds

## Abstract

The process of wound healing is complex and involves the interaction of multiple cells, each with a distinct role in the inflammatory, proliferative, and remodeling phases. Chronic, nonhealing wounds may result from reduced fibroblast proliferation, angiogenesis, and cellular immunity, often associated with diabetes, hypertension, vascular deficits, immunological inadequacies, and chronic renal disease. Various strategies and methodologies have been explored to develop nanomaterials for wound-healing treatment. Several nanoparticles such as gold, silver, cerium oxide and zinc possess antibacterial properties, stability, and a high surface area that promotes efficient wound healing. In this review article, we investigate the effectiveness of cerium oxide nanoparticles (CeO_2_NPs) in wound healing—particularly the effects of reducing inflammation, enhancing hemostasis and proliferation, and scavenging reactive oxygen species. The mechanism enables CeO_2_NPs to reduce inflammation, modulate the immunological system, and promote angiogenesis and tissue regeneration. In addition, we investigate the efficacy of cerium oxide-based scaffolds in various wound-healing applications for creating a favorable wound-healing environment. Cerium oxide nanoparticles (CeO_2_NPs) exhibit antioxidant, anti-inflammatory, and regenerative characteristics, enabling them to be ideal wound healing material. Investigations have shown that CeO_2_NPs can stimulate wound closure, tissue regeneration, and scar reduction. CeO_2_NPs may also reduce bacterial infections and boost wound-site immunity. However, additional study is needed to determine the safety and efficacy of CeO_2_NPs in wound healing and their long-term impacts on human health and the environment. The review reveals that CeO_2_NPs have promising wound-healing properties, but further study is needed to understand their mechanisms of action and ensure their safety and efficacy.

## 1. Introduction

### 1.1. An Overview of Wound Healing

Skin is the largest organ in the human body, serving as a barrier to protect the internal organs of the body from external factors [1,2]. It is the most susceptible organ that can easily get damaged due to burns, wounds, cuts, and scrapes. Despite superficial wounds, it has a remarkable ability to self-repair. However, deeper wounds and larger defects require more complex treatments to heal completely. If these wounds are not treated properly, they may lead to persistent acute and chronic health issues including infections, tissue damage, and scarring, and can sometimes be fatal. Therefore, treating wounds promptly and effectively is vital to minimize the issues and promote full healing [1,3]. Since wound healing is a complex and dynamic process involving multiple stages and cellular mechanisms to restore the tissue’s functionality and prevent infections, delayed wound healing becomes a major issue by increasing prolonged hospitalization, chronic diseases such as diabetes and cardiovascular disease, and patient quality of life [4,5]. Thus, new wound healing methods are necessary. One promising strategy uses nanotechnology because of various properties such as enhanced drug delivery, improved wound closure, reduced scarring, biocompatibility, reduced inflammation, and antibacterial properties.

Nanomaterials have recently been developed for a variety of wound-healing applications. This is due to their higher surface-to-volume ratio, which is responsible for their distinct properties. These nanomaterials exhibit outstanding bactericidal, biocompatibility, and hemostatic capabilities [6]. Due to their small size, nanomaterials’ permeability and retention effects are improved, enabling the delivery of targeted drugs and the detection of malignancies. Several nanomaterials that can enhance wound healing without any adverse effects on the body have been evaluated. Some examples include zinc oxide, silica, gold, polycaprolactone, and silver nanoparticles. They have exhibited constructive results comprehending good biocompatibility, re-epithelialization, bactericidal activity, and reduced scar formation [7]. In particular, cerium oxide (CeO_2_NPs) stands out among the numerous nanomaterials utilized for wound healing applications as one of the most promising materials due to its outstanding antioxidant, anti-inflammatory, antibacterial, and angiogenic capabilities [8].

Consequently, there is a growing interest in using CeO_2_NPs for wound healing. This review will recapitulate the current research on the use of CeO_2_NPs for wound healing and emphasize their potential advantages and disadvantages. We have focused on the characteristics and mechanisms of action of CeO_2_NP-based scaffolds and wound-healing scaffolds/materials that aid in wound healing because they offer additional antibacterial, biocompatible, and hemostatic qualities that aid in the restoration of skin integrity [9]. Despite the availability of numerous therapies for wound healing, there is a need for more effective and efficient treatments. Recent research has demonstrated that CeO_2_NPs have the potential to enhance wound healing by promoting angiogenesis and collagen synthesis while minimizing inflammation and oxidative stress.

### 1.2. Phases of Wound Healing

An optimal wound healing process consists of four phases: hemostasis, inflammation, proliferation, and remodeling. Each of these stages and their physiological endeavors are performed in a cycle for an appropriate period [6]. The first phase of wound healing pertains to eliciting hemostasis by vasoconstriction, facilitating clot formation initiated by platelet aggregation at the wound site to minimize blood loss and provide a fibrin matrix formation which aids in cell migration essential for eventual phases of wound healing. Platelets are essential in the formation of clots and also promote multiple growth factors including transforming growth factor (TGF)-β, platelet-derived growth factor (PDGF), fibroblast growth factor (FGF), and epidermal growth factor (EGF) and cytokines which are used to regulate healing cascade. This phase lasts minutes to hours and moves into the inflammatory phase [10,11,12,13].

The inflammatory phase is a protective response stage which removes invading microorganisms and cellular debris from the wound site. This is accomplished by infiltrating neutrophils, lymphocytes, and macrophages into the affected tissue. Neutrophils migrate to the injury site and destroy invading pathogens. In addition, it generates reactive oxygen species and other chemical substances that contribute to killing pathogens and recruiting other immune cells at the inflammation site. T lymphocytes are essential components of cell-mediated immunity, which is the process by which the immune system detects and destroys aberrant or infected cells in the body. The function of macrophages in the healing process is multifaceted. They play a significant role in facilitating the removal of debris, bacteria, and injured cells at the wound site. They promote angiogenesis by secreting growth factors and releasing cytokines that stimulate cell proliferation and differentiation, leading to damaged tissue healing. In addition, macrophages stimulate apoptosis and the subsequent clearance of apoptotic cells, ultimately facilitating inflammation eradication. Following the inflammatory phase, the tissue aims at achieving angiogenesis, wound closure, and re-epithelialization by the action of fibroblasts, keratinocytes, macrophages, and other cells where collagen and extracellular matrix synthesis occurs, enabling wound repair [10,11,12,14,15].

**Figure 1:**
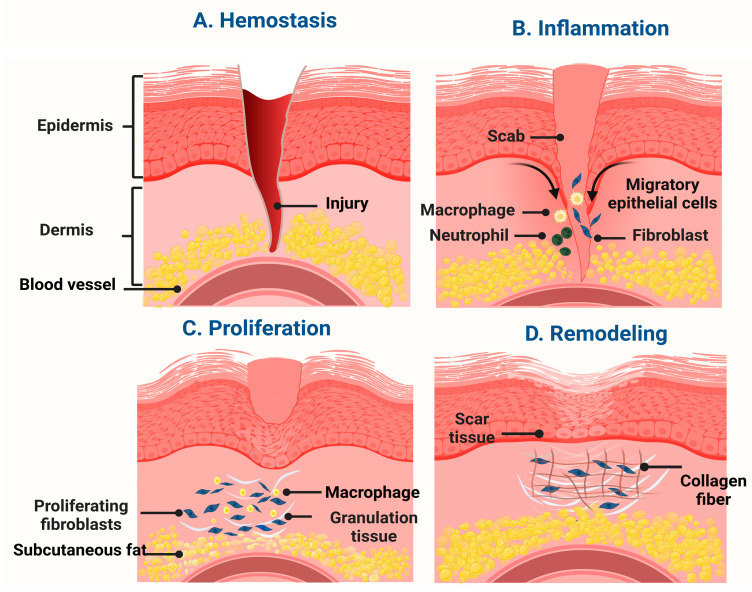
The progression of wound healing through various phases.

After the inflammatory response, the wound becomes debris-free, and the proliferation phase begins. During this phase, the injured tissues are reconstructed using a variety of cellular and molecular mechanism pathways. Fibroblasts, endothelial cells, and myofibroblasts migrate to the wound site and rebuild new tissue. Fibroblasts are responsible for producing collagen, which is an essential protein for the structural integrity of the tissue to be maintained. Endothelial cells generate new blood vessels for tissue oxygenation and nourishment. Myofibroblast cells are specialized cells that help compress the wound and bring the edges closer after injury. As the incision heals, tissue strengthens and becomes stable, which lowers the likelihood of infection. The final and longest phase of wound healing is the remodeling phase, which intends to reorganize the extracellular matrix structures to maximize the tensile strength of the newly developed tissue and is marked by the formation of scar tissue having a more parallel collagen arrangement compared to normal tissue. The main traits of this phase are the deposition of collagen, the rearranging of fibers, and the contraction of scar tissue. In addition, the rate of wound healing and its effectiveness can be determined by various factors, including the patient’s age, the type and severity of the injury, and existing health conditions (Figure 1) [11,12,16].

### 1.3. CeO_2_NPs and Their Properties Suitable for Wound Healing

CeO_2_NPs, also referred to as nanoceria, have gained attention in wound healing due to their unique properties such as antioxidant, anti-inflammatory, anti-bacterial, and pro-angiogenic properties and biocompatibility. CeO_2_NPs decrease oxidative stress, inflammation, and wound size, and promote healing. In addition, they can enhance the catalase and superoxide dismutase (SOD) enzyme activity which protects cells from damage and supports curing [17]. The antioxidant property protects cells from oxidative stress, allowing Ce^3+^ and Ce^4+^ oxidation states to act as antioxidants and pro-oxidants [18,19]. It has extensive industrial applications in medical, optical and catalysis technology. In addition, they promote new angiogenesis, stimulate endothelial for cell proliferation, and enhance oxygenation nutrient supply to the wound site.

They have been extensively used in various applications, including corrosion protection, fuel oxidation catalysis, catalysis, electrochemistry, photochemistry, fuel cells, nitric oxide radicals, chemical mechanical polishing, and biomedical applications compared to other nanoparticles [20,21]. In addition, due to their thermal stability, great mechanical properties, good oxygen storage ability, and high retention on binding enzymes [22], CeO_2_NPs can be injected into tissues to protect against a broad range of oxygen-based reactive species, enhance the redox state of the blood, and improve wound healing effectiveness both in vitro and in vivo [23].

## 2. Role of CeO_2_NPs in Wound Healing Mechanism

For effective wound healing, rapid hemostasis, proliferation, and migration of cells to the wound site, as well as rapid re-epithelialization, are necessary [6]. CeO_2_NPs are of particular interest in radical-mediated reactions. Certain applications include diabetes mellitus, a condition marked by elevated blood glucose levels and afflicted insulin-producing β cells of the pancreas. Antioxidant CeO_2_NPs drastically reduce intracellular ROS levels, preventing β-cell apoptosis; another application includes the targeted destruction of cancer cells [24].

### 2.1. Properties of Reactive Oxygen Species (ROS) Scavenging and Antioxidants

**Figure 2:**
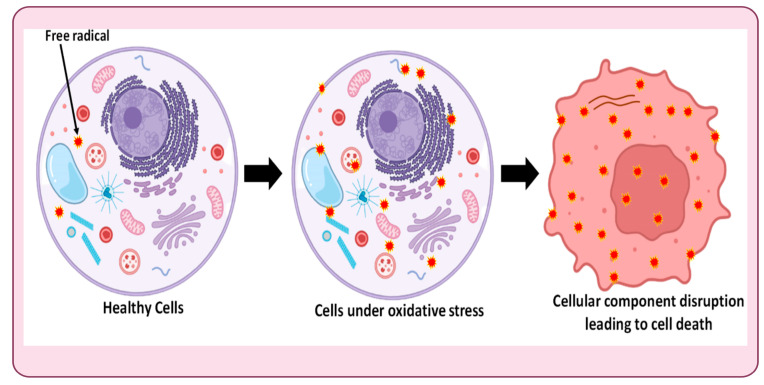
Adverse reaction of increased oxidative stress on the cells [7].

A persistent wound eventuates when it fails to progress through the different phases of wound healing and this, in turn, induces more inflammation and greater levels of reactive oxygen species (ROS), which further prolongs the healing process due to increased oxidative stress. The effect of increased oxidative stress on cells is shown in Figure 2. The formation of antioxidant systems can lower ROS levels and hence minimize oxidative stress, one of the most important aspects of wound healing [9].

**Figure 3:**
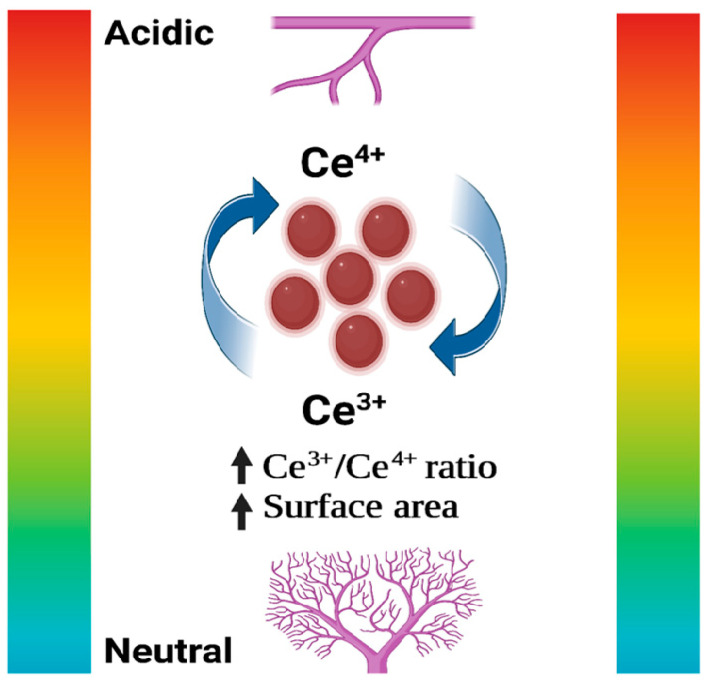
Pro-oxidant and antioxidant behavior of CeO_2_NPs with varying pH. Adapted from MDPI open access [28].

CeO_2_NPs allow ROS accumulation, exacerbating oxidative stress and ultimately leading to cancer cell apoptosis [25]. This is because they can act as either an oxidant or an antioxidant depending on the pH of the surrounding environment, which is depicted in Figure 3. The pH of cancer cells is acidic relative to the normal cells, prompting the pro-oxidative effect of CeO_2_NPs [25,26]. They also have a therapeutic effect on cerebral ischemia, a condition developed due to insufficient blood flow to the brain, resulting in oxygen deprivation in the cells and cell death. To restore normal blood flow, oxygen consumption is reduced in the mitochondria of neural cells, and this quick shift in oxygen levels results in the conversion of excessive oxygen into ROS and RNS (reactive nitrogen species). CeO_2_NPs are employed to reduce oxidative stress because of their antioxidative properties, and they also stimulate neurogenesis [24,27].

CeO_2_NPs were found to improve vascularization through their angiogenic properties, where CeO_2_NPs promoted the formation of new vessel networks by regulating intracellular oxygen and stabilizing vascular endothelial growth factor (VEGF) and Hypoxia-inducible factor-1 (HIF-1), which are pertinent in angiogenesis, cell proliferation, and other processes [29,30]. CeO_2_NPs can self-renew because they have valences of +3 or +4 allowing flexibility to switch between anti- and pro-oxidant states [19]. Due to this transition, they can switch between antioxidant and pro-oxidant capabilities, with pro-oxidant properties predominating at lower pH values [31]. CeO_2_NPs pro-oxidant nature allows them to cause oxidative stress, which could be beneficial in cancer treatment. Cancer cells, unlike normal cells, have a redox imbalance and produce abundant reactive oxygen species (ROS). The pro-oxidant activity of CeO_2_NPs is particularly important in this setting because the pH of tumor cells is typically decreased, allowing the pro-oxidant system to increase oxidative stress and eventually cause cell apoptosis [32], which would otherwise be hazardous in the biological environment [21]. 

**Figure 4:**
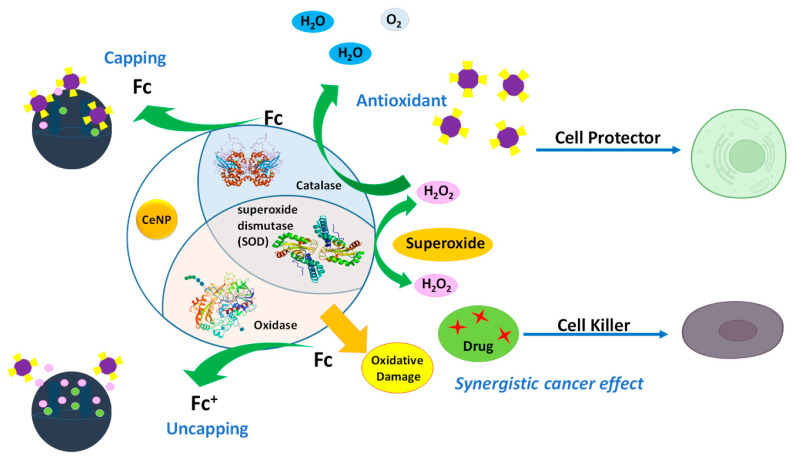
Implication of CeO_2_NPs in anticancer and cell recovery mechanisms. Adapted from MDPI open access [38].

CeO_2_NPs anti-oxidant characteristics, on the other hand, enable ROS scavenging by catalyzing the breakdown of ROS. Figure 4 demonstrates the pro-oxidant and antioxidant responses of CeO_2_NPs. Bone regeneration applications are one of the instances where CeO_2_NPs antioxidant potential is employed. In the case of a fracture or surgical wound, oxidative stress on the bone increases, resulting in the formation of free radicals, which might eventually lead to bone apoptosis. CeO_2_NPs can aid in eradicating these free radicals, enhancing biomineralization and overall mechanical characteristics [33]. The smaller Ce^3+^ particles boost this catalytic activity of ROS scavenging [34], maintaining the balance between ROS formation and destruction [35]. Therefore, the therapeutic strategy should direct CeO_2_NPs synthesis to minimize adverse body reactions [21]. Furthermore, higher Ce^4+^ ion concentration increases catalase mimetic activity, but higher Ce^3+^ ion concentration increases superoxidase dismutase (SOD) mimetic activity [36]. Catalase and SOD are antioxidant enzymes, which may be one of the fundamental mechanisms of CeO_2_NPs ROS scavenging abilities [37]. These characteristics make it a promising material for use in wound healing applications.

Another important aspect of the CeO_2_NPs is the therapeutic effects observed in the CeO2 NPs–microRNA (miR146a) conjugate. MicroRNAs are small noncoding RNA molecules that are significant to wound healing mechanisms. MicroRNAs, specifically miR146a, play a significant role in the regulation of inflammatory immune responses of the body by modulating the key inflammatory signals critical for wound healing [34,38,39,40,41,42]. In persistent chronic wounds, miR146a dysregulation may occur, hindering the wound healing procedure and requiring its supplementation to enhance the process [38,42]. However, miR146a cannot be administered directly due to its high degradability and the negative surface charge that causes electrostatic repulsion with the cell membrane, lowering cellular uptake and therapeutic efficacy. Therefore, they are generally conjugated with other inorganic nanoparticles such as silica, cerium oxide, and gold nanoparticles to stabilize and neutralize the negative charge and enhance the target delivery to the cells [34,38,43,44]. CeO2NPs are one of the most considered materials for conjugation with miR146a, as reports have suggested better cell targeting and reduced oxidative stress on the gene, ensuring improved delivery of miR146a to the targeted site and contributing to rapid wound healing [38,43,45]. The study conducted by Dawberry et al. found that administering a combination of CeO_2_NP (divalent metal oxides that scavenge free radicals) and miR146a (which controls the proinflammatory NF-B pathway) promotes diabetic wound healing. The study involved conducting immunohistochemistry and gene expression studies to determine how CeO_2_NP-miR146a promotes diabetic wound healing. The findings suggest that intradermal injection of CeO_2_NP-miR146a accelerates the healing of diabetic wounds by increasing wound collagen, promoting angiogenesis, reducing inflammation, and lessening the effects of oxidative stress. This is achieved by targeting the NF-B pathway and reducing oxidative stress, creating a wound environment favorable to angiogenesis and collagen production [46].

### 2.2. Effects on Reducing Inflammation and Modulating the Immunological System

The anti-inflammatory and immunomodulatory actions of a material pertain to its capability to alter the immune system’s response and diminish inflammation. Prolonged inflammation can lead to tissue damage and facilitates the emergence of diverse ailments, such as autoimmune disorders, cancer, and cardiovascular diseases. Inflammation is a normal immune system reaction to an injury or infection. Proinflammatory cytokines and chemokines, which stimulate immune cells to relocate to the site of inflammation, are inhibited when anti-inflammatory medications are used [47]. On the other hand, depending on the condition, immunomodulatory agents can either boost the immune system’s reaction or suppress it. Because of their valence and oxygen defect properties, CeO_2_NPs (nanoceria) may be autoregenerating free radical scavengers. The enzyme inducible nitric oxide synthase (iNOS) overproduces the free radical nitric oxide (NO), a major mediator of inflammation. A study by Suzanne et al. reported CeO_2_NPs scavenge ROS or free radicals and inhibit inflammatory mediator production in macrophages. They performed an in vivo study showing CeO_2_NPs deposition in mouse tissue without pathogenicity. A novel therapy for chronic inflammation could also be derived from nanoceria since it can reduce ROS production in states of inflammation [48].

Another study aiming to investigate the anti-inflammatory effect of CeO_2_NPs was reported. The primary objective was to investigate macrophage phenotype, cytokine expression, and proliferation under chronic and acute inflammatory conditions, and osteoinduction and differentiation of human mesenchymal stem cells (hBMSCs). They observed both acute and chronic conditions, iNOS activity decreased, and there was a significant increase in the anti-inflammatory cytokines gene in chronic inflammatory conditions. hBMSCs cultured without osteogenic media showed no increase in alkaline phosphatase (ALP) activity or mineral deposits, but a significant increase in calcium deposits, ALP activity, and osteogenic-related genes was expressed when CeO_2_NPs and osteogenic media were both supplemented [49].

Most NPs are captured by the spleen and liver irrespective of their dose difference, surface state, morphology, and aggregation. NPs clearance rate and cellular/tissue distribution in the body depend on NPs characteristics [50]. There is a need to develop new tools and concepts to understand the full potential and avoid the risk associated with NPs, as their pharmacokinetics significantly differ from traditional small molecules. A study demonstrated the long-term effect of CeO_2_NPs inside the body. They administrated 3 nm nanoceria at a concentration of 5.7 mg/kg body weight through an intravenous route in healthy mice. The biodistribution of CeO_2_NPs at different time points was measured in different organs such as the liver, brain, spleen, kidney, lungs, and brain, and in fecal and urinary excretion. They reported CeO_2_NPs accumulation in the spleen and liver, and their decay was exponential, with 50% of NPs excreted in 100 days. CeO_2_NPs does not damage any target organs; no weight loss or apathy was observed in experimental rats [50].

### 2.3. Facilitation of Angiogenesis and Tissue Regeneration

Angiogenesis is the process through which new blood vessels develop, assisting in the repair and restoration of damaged tissue, making it one of the most critical wound-healing elements [51]. There are several mechanisms to promote angiogenesis, one of which includes the use of growth factors, such as vascular endothelial growth factor (VEGF), that act as stimulants for blood vessel formation [52]. It also a play key role in delivering oxygen and nutrients to injury sites. Many studies have also shown that nanoparticles can stimulate angiogenesis by triggering certain signaling pathways involved in the development of vascular networks; among them are gold and mesoporous silica nanoparticles. Another approach is to employ stem cell therapy, which entails the differentiation of stem cells into various cell types that aid in the development of blood vessels [53]. Therefore, these are some of the techniques that may be incorporated to facilitate angiogenesis, thereby promoting tissue repair and regeneration and expediting the process of wound healing.

## 3. Potential Applications of CeO_2_NPs in Wound Healing

In biomedical engineering, polymers are frequently used to extend the application scope and enhance material characteristics. Polycarbonate and acrylic polymers are utilized in dentistry. PCL fibers are ideal for wound dressings and medication delivery [39]. Polymeric coatings are typically used for medical implants to increase hemocompatibility and prevent corrosion [42]. Due to their biomimetic properties, polymeric materials are also utilized as tissue regeneration scaffolds. Polymers can serve as matrices for encapsulating several nanoparticles, medications, and other substances, and as simple support structures [40,54]. Therefore, integrating CeO_2_NPs with other polymeric materials or nanofibers can broaden its application to wound healing by allowing for longer drug release at the wound site and accelerating the wound healing process, improving overall efficiency. Hereby, we have discussed a few recent and most relevant CeO_2_NP-conjugated polymeric scaffolds that have shown potential in wound healing applications.

### 3.1. CeO_2_NP-Incorporated PHBV Membranes

**Figure 5:**
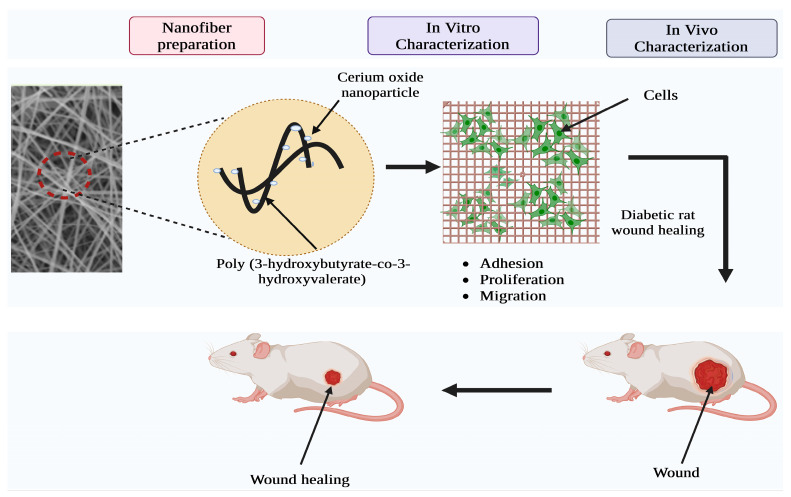
PHBV membrane with CeO_2_NPs for diabetic wound healing in vivo [34].

PHBV (poly(3-hydroxybutyrate-co-3-hydroxyvalerate)) is a biodegradable, nontoxic, and biocompatible polyhydroxyalkanoate-type polymer (Figure 5) [41]. PHBV cell membrane-encapsulated CeO_2_NPs have shown potential for treating diabetes-related wound healing complications. Such membranes were shown to have the ability to repair wounds in an experiment conducted on living animals using diabetic rats. PHBV membrane-based wound dressing matrices have been reported to enhance cell proliferation, migration, and angiogenesis. The electrospinning approach was utilized to fabricate nanofibers based on PHBV–cerium oxide nanoparticles. This method requires the preparation of PHBV solutions that include various amounts of CeO_2_NPs in a 9:1 ratio of chloroform to dimethyl formamide (DMF). Continuous nanoscale fibrous membranes were formed when a high voltage direct current (DC) power source was supplied, and these membranes were subsequently deposited on the collector [34]. The electrospinning approach produced highly porous nanofiber matrices that allowed oxygen and nutrients to diffuse freely, enabling quick and successful wound healing [43,55,56]. However, one significant disadvantage of this approach is the detrimental repercussions of DMF, a polar aprotic solvent, on vital organs, particularly the liver [57,58,59]. This can be mitigated by using a membrane distillation process, in which the vapor molecules are only permitted to travel over a hydrophobic membrane, eliminating any potential contaminants [60].

### 3.2. Cerium Oxide Nanoparticle-Containing Genipin Crosslinked Gelatin Hydrogel Composite (G-CeO_2_NPs)

**Figure 6:**
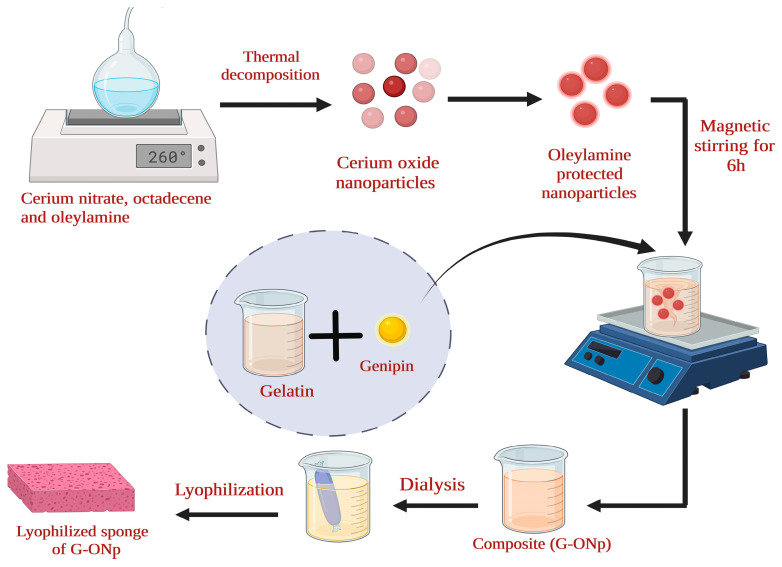
Fabrication of a gelatin hydrogel composite with cerium oxide nanoparticles (G-CeO_2_NPs) and crosslinking between the two using genipin [63].

Gelatin matrices are porous, allowing cells to move freely and providing mechanical and structural support for new tissue formation [38]. Their high biodegradability and biocompatibility have made them popular in wound healing applications. However, they cannot be used alone in biomedical applications due to their poor mechanical and antimicrobial properties and tendency to dissolve below 29 °C. Crosslinking with other polymers could improve these mechanical, bactericidal, and thermal properties [44,45,61,62]. Genipin was chosen as the crosslinking agent because of its high biocompatibility and ability to produce a stable crosslinked polymer [45,63]. It was found that the G-CeO_2_NPs composite cured wounds better than gelatin alone. In an in vivo investigation on rats, the G-ONPs composite exhibited rapid leukocyte movement and high collagen deposition rates, speeding up the wound healing process compared to other gelatin groups. For synthesizing CeO_2_NPs, the thermal decomposition method was utilized to decompose metal precursors in the presence of oleylamine, which acts as stabilizing agent to control the shape, size, and dispersion of resulting nanoparticles. Oleyalmine serves both as a reducing agent and a stabilizer, facilitating the thermal decomposition and formation of nanoparticles. To synthesize composite material, gelatin stock solution was mixed with CeO_2_NPs and then magnetically agitated. After that, genipin was added to the mixture to help generate crosslinks between gelatin and CeO_2_NPs [63] (Figure 6).

### 3.3. PVA/Chitosan-Incorporated Green-Synthesized Wound Healing Hydrogel

Polyvinyl alcohol (PVA) hydrogels are one of the most widely used polymeric materials for wound-healing applications due to their nonadherent nature and ease of removal from the wound [64]. Due to the high water content of hydrogels, they have sufficient flexibility and elasticity to fit the contour of the wound, making them an ideal material for wound dressings. However, because PVA alone has limited mechanical strength, composite hydrogel membranes are being fabricated to improve their usage in wound healing applications [65]. An article by Kalantari et al. reported synthesizing and evaluating a polyvinyl alcohol/chitosan (PVA/chitosan) hydrogel for wound healing applications. The hydrogel was incorporated with green-synthesized cerium oxide nanoparticles (CeO_2_NPs) using an extract from Zingiber officinale as the reducing, capping, and stabilizing agent. The PVA/chitosan/CeO_2_NPs hydrogel was developed using the freeze–thaw method with 0 to 1% (wt) 5 nm CeO_2_NPs as the active ingredient. Compared to the control group, the results showed that the hydrogels that included 0.5% CeO_2_NPs showed superior antibacterial activity after just 12 h. Hence, these chitosan/PVA hydrogels combined with CeO_2_NPs might be a promising contender as a strong wound dressing agent that may significantly diminish wound infections without resorting to antibiotics. This would be a significant advancement in treating chronic wounds [66].

### 3.4. PLA/PVA/PLA Trilayer Nanofibers with CeO_2_NPs

**Figure 7:**
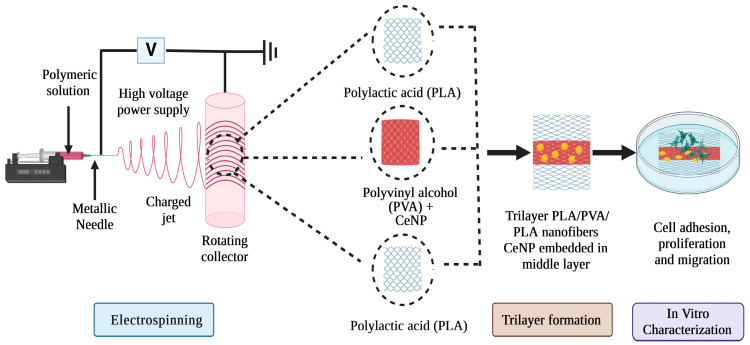
Fabrication of trilayer PLA/PVA/PLA nanofibers with CeO_2_NPs [72].

Polylactic acid (PLA) is a synthetic polymer obtained from natural sources widely employed in biomedical applications. It is one of the most prominent materials in the wound healing industry because of its exceptional mechanical and biocompatibility features [67,68]. PVA can absorb exudates and keep the wound moist. However, it cannot be used alone as a wound dressing due to its lack of bioactivity [69]. To aid in the healing of chronic wounds, Polylactic acid (PLA) and Polyvinyl alcohol (PVA) nanofibers (NFs) are sandwiched to produce a trilayer with CeO_2_NPs integrated into the nanofibers. Because PVA has a greater degradation rate, which could lead to ineffective and quick CeO_2_NPs release, it is coated with PLA, which has a lower degradation rate and assures a more regulated release of CeO_2_NPs from the inner PVA layer. This sustained release helps maintain the precise concentration of CeO_2_NPs in the wound, ensuring consistent and uninterrupted drug release for patients who require long-term treatment. The material’s overall mechanical and biocompatibility qualities are also improved, making it more effective than monolayer polymer membranes loaded with CeO_2_NPs [70]. The CeO_2_NP-loaded PLA/PVA/PLA trilayer NFs were prepared using the electrospinning method (Figure 7). Electrospun fibers exhibited high porosity, wettability, and resemblance to the natural extracellular matrix, promoting cell proliferation and migration and enhancing wound healing [71].

The outer layer of PLA was deposited on the collector following the electrospinning process. The same process was utilized to produce the middle layer, the CeO_2_NP-loaded PVA for a substantially longer length due to the greater thickness. The PLA solution was electrospun for the same length as the first layer to produce the third layer. The fibrous membranes deposited on the collector were carefully removed and stored at room temperature in a desiccator [72].

### 3.5. Polycaprolactone–Gelatin Nanofiber with CeO_2_NPs Functionalization (PGNPNF)

**Figure 8:**
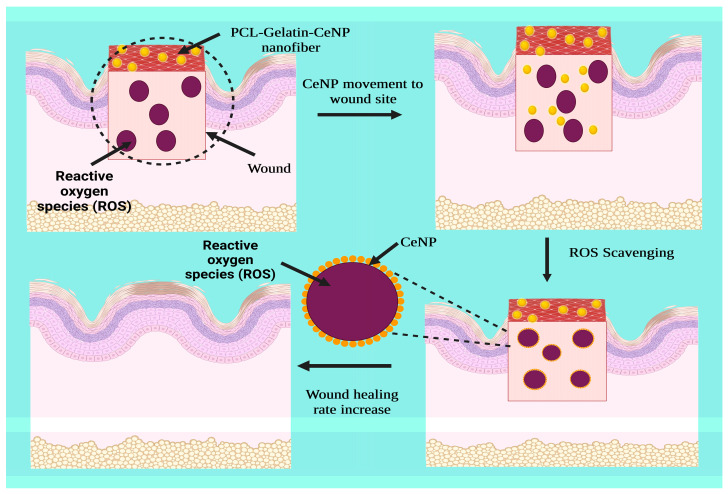
Wound healing via ROS scavenging using CeO_2_NPs loaded PCL-gelatin nanofibers [57].

PCL is a synthetic polymer material exploited for wound-healing applications due to its exemplary biological and mechanical properties [73]. However, it could not be used aloneso due to its poor adhesivity. As a result, gelatin, a highly biodegradable and biocompatible polymer with exceptional nanofiber-forming capabilities, was employed in conjunction with PCL to aid in wound repair [60,69,71]. Nanofibers fabricated exhibited similar-to-optimal scaffolds as they offered greater surface area, porosity, and permeability, encouraging cell adhesion and proliferation [71]. Various investigations revealed that the PCL–gelatin nanofiber combination outperformed pure PCL regarding cell proliferation [60]. This composite was a support structure for the CeO_2_NPs, which had effective ROS scavenging properties [57]. PCL–gelatin nanofiber loaded with CeO_2_NPs was fabricated using the electrospinning process; 10% *w*/*v* of PCL and 20% *w*/*v* gelatin were combined in the hexafluoroisopropanol (HFIP) solvent for about 10 h, followed by 25% *v*/*v* of the 20 mM CeO_2_NPs suspension. This polymeric solution was made to discharge in the form of nanofibers over an aluminum foil [58]. The considerable decrease in PCL crystallinity that occurred as a result of gelatin blending showed a significant effect on the degradation behavior of the scaffold. SOD mimetic tests showed that the dissolution of uncrosslinked gelatin permitted the quick release of nanoparticles, whereas the use of PCL guaranteed that the structural integrity of nanofibers was maintained. In addition, the SOD mimetic activity demonstrates that PGNPNF has an antioxidant impact in a variety of buffer systems. In addition, the ability of PGNPNF to protect cells from the oxidative damage caused by Hydrogen peroxide is more evidence that the compound possesses antioxidant and cell protection properties. As a result, PGNPNF meshes, which have the potential to act as antioxidants, might be investigated for use as a wound dressing material (Figure 8).

### 3.6. Curcumin and CeO_2_NP-Integrated Dextran-Based Amphiphilic Nanohybrid Hydrogel System

**Figure 9:**
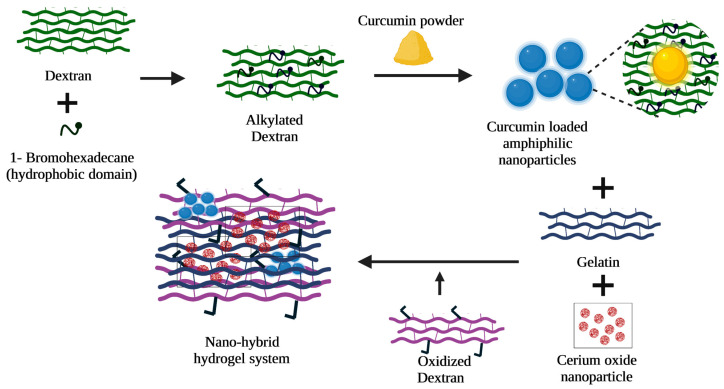
Synthesis of curcumin and CeO_2_NP-integrated Dextran-based amphiphilic nano-hybrid hydrogel system [64].

Curcumin promotes rapid wound healing due to its excellent anti-inflammatory, antimicrobial, and antioxidant nature. Additionally, it had a greater ability to enhance tissue formation and remodeling [59]. However, it had limited applications due to its poor bioavailability and aqueous insolubility. Therefore, curcumin was integrated with 1-bromohexadecane-dextran, which acted as a carrier system for the curcumin formulations. Additionally, it was said to overcome the drawbacks of curcumin when administered as a primary wound-healing agent. In addition to curcumin, CeO_2_NPs were also incorporated, and delivered at the wound site to reduce inflammation and oxidative stress, thus promoting cell proliferation for rapid wound healing. The curcumin and CeO_2_NP-integrated O-hexadecyl-dextran were prepared using the freeze-drying method. The O-hexadecyl-dextran (HD) was prepared by dissolving dextran, NaOH, and 1-bromohexadecane-dextran in tetrahydrofuran (THF). The solution was then dialyzed and freeze-dried. The HD particles obtained were optimized by dissolving in Milli-Q-water followed by freeze-drying. The curcumin–acetone solution was then dropped into the previously produced solution, and the resulting mixture was lyophilized. The obtained curcumin-encapsulated nanoparticles and CeO_2_NPs were added to the gelatin solution to form the nanoparticle–hydrogel integrated system [64]. The hydrogel showed regulated and extended drug release and increased cell migration, and provided antioxidant and in vivo anti-inflammatory action. The study emphasizes the need to combine hydrogel and nanosystems to improve wound healing, drug transport, bioavailability, cytotoxicity, and therapeutic effects. Based on the promising results, more in vivo research is needed to translate its potential for quick wound healing into practical applications (Figure 9). Decellularized extracellular matrix (ECM) has been widely used for wound healing. However, ECM failed to integrate tissue and restore the tissue function properly when elevated levels of free radicals and biofilm formation occur at the wound site. To address this issue, a nanoemulgel system (DG-SIS/Ce/NC) was developed by combining nanoceria, curcumin nanoemulsion, and goat small intestine submucosa ECM gel. These nanoemulgel systems comprise decellularized ECM of caprine small intestine submucosa (DG-SIS), curcumin encapsulated eucalyptus oil-based nanoemulsion (Ce), and nanoceria (NC). The resulting formulation exhibited antibacterial, antioxidant, hemocompatible, biocompatible, and enhanced wound-healing properties. The DG-SIS/Ce/NC formulation showed the highest free radical scavenging capacity, sustained curcumin release, good skin permeability, increased cell proliferation, full-thickness wound contraction, and collagen synthesis. The enhanced wound healing is expected due to the synergistic effect of Ce, NC and DG-SIS. The reports have shown that DG-SIS/Ce/NC formulation holds potential for use as a nanoemulgel system in full-thickness wound healing applications [74].

### 3.7. Gelatin Methacryloyl Hydrogel Patch with CeO_2_NPs

Gelatin methacryloyl (GelMA) hydrogels are used in wound healing due to their hydrogel-like nature, allowing them to retain moisture and biocompatibility with the surrounding tissues [65]. Nevertheless, their usage is limited due to nonadhesiveness and poor mechanical strength [75]. However, they could be used conjointly with CeO_2_NPs, as this allowed for a combined benefit of free radical scavenging activity of CeO_2_NPs and controlled delivery of the drug at the injured site which otherwise would result in a rapid, excessive release of CeO_2_NPs which might turn out to be toxic. The CeO_2_NP-incorporated GelMA hydrogel patch was synthesized using ultrasonication wherein CeO_2_NPs were mixed with GelMA solution and crosslinked under UV irradiation. The resulting patches were lyophilized for roughly two days after being rinsed with demineralized water [75]. The findings of the study on wound healing demonstrated that diabetic rats treated with patches loaded with CeO_2_NPs exhibited significantly improved wound healing. The findings as a whole suggest that CeO_2_ NP-loaded GelMA hydrogels are among the most promising materials for the development of therapeutically applicable patches for the treatment of diabetic wounds.

### 3.8. CeO_2_NP Nanocomposite Hydrogels

**Figure 10:**
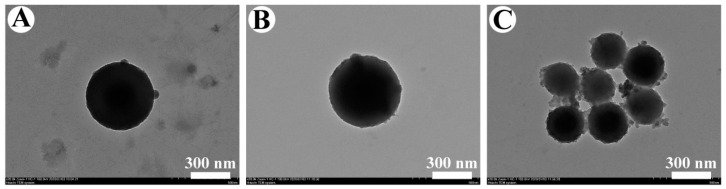
Representing TEM images of CeO_2_NPs–Bioglass nanocomposites i.e. (**A**) shows TEM images of 0 CeO_2_NP-BG, (**B**) 2 CeO_2_NP-BG and (**C**) 5 CeO_2_NP-BG. Adapted from MDPI open access [78].

Direct contact with several biomaterials, such as bioglass, can cause the biomaterial to adhere to the wound bed, which can lead to laceration and other negative wound responses [76]. Hydrogel is a type of hydrophilic polymer that naturally has a three-dimensional structure and has the potential to release therapeutic compounds as an appealing option to many other kinds of system techniques [77]. Hydrogels filled with cerium oxide nanoparticles, bioglass, or other components can prevent unfavorable responses. Both natural and synthetic biomaterials, such as alginate, collagen, and chitosan, can be utilized to produce hydrogels. In a study conducted by Chen et al., the researchers decided to employ photocrosslinkable and biodegradable gelatin methacryloyl (GelMA) as the backbone of the hydrogel. The study developed a multifunctional injectable composite hydrogel by combining cerium-containing bioactive glass with Gelatin methacryloyl hydrogel. The resulting hydrogel was cytocompatible, promoted endothelial cell migration and tube formation, and exhibited excellent antibacterial properties. In vivo studies on diabetic rats demonstrated that the hydrogel significantly improved wound healing by accelerating granulation tissue formation, collagen deposition, and angiogenesis. The study suggests that developing multifunctional materials with antibacterial and angiogenic properties can promote the repair of diabetic wound healing [78]. Recently, Gong et al. developed an injectable self-healing ceria-based nanocomposite hydrogel with ROS-scavenging activity to speed up wound healing. The dynamic Schiff base reaction crosslinked polyethyleneimine-coated cerium oxide nanorods with benzaldehyde-terminated F127 (F127-CHO) to create nanocomposite hydrogels (FVEC hydrogel). The FVEC hydrogel was thermosensitive, injectable, self-healing, and ROS-scavenging. It was biocompatible and biodegradable and improved wound healing and epithelial regeneration in full-thickness skin wound tests in mice. The study proposes a multifunctional CeO_2_NP-based nanocomposite hydrogel for wound healing and regeneration, which removes ROS and accelerates wound closure and tissue regeneration (Figure 10) [79].

Ma et al. conducted a study where they designed a multifunctional wound dressing possessing antibacterial properties, with the aim of expediting the process of wound healing. They prepared novel monodispersed sodium alginate–carboxymethyl chitosan (SA-CMCS) spheres at different ratios via crosslinking agent Ce^3+^. The antibacterial potential of the system is made more appealing to Staphylococcus aureus (*S. aureus*) and Escherichia coli by the progressive release of Ce^3+^ from the spheres (*E. coli*). In addition, the combination of SA and CMCS bestows the spheres with high biocompatibility, strong stability, and the capacity to speed up the healing process of wounds. The Electrospray method was utilized to generate Ce^3+^-crosslinked SA-CMCS spheres. These spheres can enhance wound healing and inhibit bacterial growth. The electrostatic spray approach was successfully used to generate Ce^3+^-crosslinked SA-CMCS spheres of millimeter size, displaying good antibacterial characteristics and effectively promoting wound healing. During the spinning process, various ratios of SA to CMCS were optimized. Among all, the SA-CMCS-1 spheres, out of all the SA-CMCS spheres, are the most stable, and their antibacterial power is the greatest due to the optimum Ce^3+^ release rate. This helps to prevent wound infection while the wound is healing. In addition, based on the high biocompatibility and wound-healing-encouraging effect of SA and CMCS, the SA-CMCS spheres, particularly the SA-CMCS-1 spheres, also demonstrate the capacity to enhance wound healing [80].

## 4. Discussion

Various scaffolds are being developed to assist in wound healing and tissue regeneration. Scaffolds of various biomaterials, such as polymers and metals, create wound-healing environments. Porosity, flexibility, biocompatibility, appropriate mechanical strength, and others are the qualities of scaffolds that promote quick wound healing [55,81]. Cerium oxide nanoparticles have been demonstrated as a potential material for addressing various issues and serving as antibacterial agents, redox elements, bioscaffolds, free radical scavengers, cancer treatment agents, biofilm inhibitors, and in many more biomedical applications. In addition, the fabrication of these nanoparticles via either physical, chemical, or biological methods may also assist in various fields, including solar cells, chemical mechanical polarization, and catalysis for fuel oxidation [82]. A few comparisons of various cerium oxide-based wound healing scaffolds are listed in Table 1. Dhall et al. discussed a novel method for fabricating cerium oxide nanoparticles, including a green synthesis method that provides safe routes to prepare CeO_2_NPs, that possess advantages, such as low cost, biocompatibility, enzyme mimetic activities, ROS scavenging activities, and others [21].

CeO_2_NPs are mainly used in biomedical applications because of their unique catalytic capabilities, low toxicity, bactericidal properties, excellent biocompatibility, and other factors. In this review, we discussed various CeO_2_NP-based scaffolds for wound healing applications, where CeO_2_NPs were combined with polymeric materials and hydrogels to improve their properties. Overall, the findings imply that CeO_2_NP-based composite scaffolds outperform hydrogels or other polymers used independently. As a result, the construction of such scaffolds would represent a significant advancement in treating chronic wounds. Chronic wounds are characterized by poor wound healing, which the development of a biofilm can trigger. The formation of a biofilm takes place when bacteria stick to the wound surface and begin to reproduce. This film secretes a glue-like matrix that protects the microorganisms within it from antimicrobial agents and the patient’s immunological response. As a result, avoiding biofilm formation is crucial [83]. In addition, biofilm may result from underlying diseases, such as diabetes, vascular disease, neuropathies, immunological inadequacy, and other similar conditions [84]. Understanding the pathophysiology of different types of wounds can lead to more accurate treatment techniques and, eventually, faster healing rates, leading to more successful wound treatments. Chronic wounds can be prevented from becoming infectious by practicing good wound care.

## 5. Conclusions

Wound healing is a complex process comprising hemostasis, inflammation, proliferation, and remodeling, and involving the coordination of various cellular and molecular mechanisms. Many factors can influence the healing process, including bacteria, infection, oxygenation, stress, foreign materials, and inflammation. Cerium oxide-based scaffolds have been shown to effectively combat these factors, leading to improved wound healing outcomes. These scaffolds have desirable properties, including anti-inflammatory and antibacterial effects against a wide range of pathogenic bacteria, including methicillin-resistant Staphylococcus aureus (MRSA), and the ability to promote tissue regeneration effects. This is achieved through the activation of various cellular and molecular pathways, including the promotion of angiogenesis, the activation of fibroblasts, and the stimulation of collagen synthesis. Despite the CeO_2_NPs possessing promising results, there are some challenges associated with their utilization, i.e., poorly established synthesis techniques, which makes it difficult to determine the size, shape, and dosage of CeO_2_NPs for wound healing. Although the antioxidant, anti-inflammatory, and antibacterial properties of CeO_2_NPs are well established, the exact mechanisms of action information are very limited. High-concentration dosage and long-term exposure may lead to toxicity. Regulatory approval is essential for product safety, and the manufacturer should adhere to predefined rules. Therefore, more research is needed to fully understand the mechanisms behind cerium oxide’s effects on wound healing. Cerium oxide-based scaffolds have become attractive for promoting wound healing in infected wounds. Further, these scaffolds can be utilized in clinical applications after optimizing and determining their effectiveness in different types of wounds and patient populations.

## Figures and Tables

**Table 1:** Comparison of various characteristics of cerium oxide-based scaffolds for wound healing.

Cerium Oxide-Based Scaffolds	Fabrication Techniques	Advantages	Limitations	Applications	References
Polycaprolactone (PCL)–gelatin nanofiber with CeO_2_NPs functionalization (PGNPNF)	Electrospinning	Cell viability and proliferation were improved. It reduced oxidative stress by lowering the number of reactive oxygen species (ROS).	Sustained delivery was not achieved because PCL breakdown was faster resulting in a rapid release of nanoparticles.	Wound dressings	[57]
Curcumin and CeO_2_NP-integrated Dextran-based amphiphilic nanohybrid hydrogel system	Freeze drying	Medication release was controlled and delayed. Cell migration and proliferation were enhanced which aided in the forming a new vascular network.	It was challenging to entrap hydrophobic curcumin inside hydrophilic nanoparticles. Despite a greater rate of disintegration at first, the hydrogel did not disintegrate and was retained.	Drug delivery	[64]
Gelatin methacryloyl hydrogel patch with CeO_2_NPs	Ultrasonication	The porous structure promoted cell mobility and proliferation. Excellent tensile mechanical characteristics and fluid retention capacity Exudate from the wound surface was absorbed effectively.	During wound healing, a fibrotic scar developed, disrupting normal tissue arrangement. Its long-term health consequences on humans are unclear.	Wound-healing patch for swift diabetic wound healing and chronic ulcer treatment.	[75]
PHBV membranes incorporating CeO_2_NPs	Electrospinning	It enhanced blood vessel development by promoting cell proliferation and adhesion.	Reduced thickness of the formed epidermal layer.	Wound dressings	[34]
Gelatin and cerium oxide nanocomposite	Magnetic stirring	Ultra-small holes enable cell migration, nourishment, and oxygen exchange, accelerating wound healing.	To generate a membranous structure, an extra crosslinking agent was needed [26].	Wound dressings	[45]
Polyvinyl alcohol nanogels with CeO_2_NPs	Freeze- thawing	Its high elasticity enabled it to wound easily. Improved fluid retention capacity, which allowed for speedier wound healing.	Maximum strength was reduced.	Wound dressings	[66]
PLA/PVA/PLA tri-layer nanofibers (NFs) loaded with CeO_2_NPs	Electrospinning	Cell adhesion, growth, and proliferation were all improved. Improved biocompatibility and mechanical characteristics. This allowed for a more steady and long-lasting release of the drug.	A high amount of CeO_2_NPs was needed to generate a more stable trilayer.	Drug delivery	[72]

## Data Availability

Data sharing not applicable since no new data were generated.
